# Magneto-Electroluminescence in ITO/MEH-PPV:PEO:LiCF_3_SO_3_/Al Polymer Light-Emitting Electrochemical Cells

**DOI:** 10.3390/mi10080546

**Published:** 2019-08-17

**Authors:** Mingpeng Zhu, Xueting Yuan, Gang Ni

**Affiliations:** 1 Department of Optical Science and Engineering, Fudan University, Shanghai 200433, China; 2 Shanghai Engineering Research Center for Ultra-Precision Optical Manufacturing, Fudan University, Shanghai 200433, China

**Keywords:** magnetic field effects, light-emitting electrochemical cell, organics semiconductor

## Abstract

Magnetic field effects (MFE) have been extensively studied in organic light emitting diodes because of their potential application in organic spintronics devices. However, only a few studies on MFE in organic light-emitting electrochemical cells (LEC) have been reported. In this paper, magnetic field effects on the electroluminescence of an LEC device with the structure of ITO/MEH-PPV:PEO:LiCF_3_SO_3_/Al were studied at various temperatures. The luminance–current–voltage curves of the device shows the typical bi-polar characteristics of LECs; positive magnetic electroluminescence (MEL) was observed with a value of about 2.5% (B = 42 mT, 250 K), showing a Lorentzian line shape. With a decrease in temperature, the MEL value and the threshold voltage increased accordingly, below the possible mechanism is discussed.

## 1. Introduction

Recently, spin-related physical phenomena in organic semiconductors have been widely studied. Magneto-electroluminescence (MEL)—that is, a change in the electroluminescence upon application of a magnetic field, has been observed in non-magnetic organic light emitting diodes (OLEDs), the MEL values and signs depend on the organic materials, applied voltage, and temperature [1,2,3,4,5]. With a decrease in temperature, the change of MEL shows divergence, e.g., it decreases for Alq_3_-based devices [6] and increases for MEH-PPV based OLEDs [7]. The effect has been used as a tool to reveal the intrinsic spin-related process, such as spin-orbit coupling and hyperfine interaction in organic semiconductors [8]. However, the mechanism of MEL is still uncertain, several competing models, such as an exciton model, a bipolaron model, an exciton polaron interaction model and a triplet-triplet annihilation model, have been studied to try to explain it [2,9,10,11].

The light-emitting electrochemical cells (LECs) were introduced by Pei in 1995, providing an alternative approach to light emitting from organic semiconductors [12]. By doping conjugated polymers with ion-transport materials, upon an applied electric field, electrochemical redox and the redistribution of ions, cause p-type doping near the anode and n-type doping near the cathode, forming the p–i–n junction in the emissive layers and then emitting the light [13]. Due to the air-stable cathode and simple device structure, the LECs become a possible alternatives of OLEDs for industrial processing in the future. Recently, the device performance of the LECs have greatly improved through better device and material engineering [14]. Until now, only few studies on MEL in LECs have been reported [15,16,17]. In the LECs, the mobile ions play an important role in the electroluminescence process, and showing temperature-dependent conductive behavior [18]. However, an MEL in LECs at low temperatures has never been reported. In this paper, we investigated the temperature dependence of MEL in MEH-PPV based LECs, and discussed the possible mechanism.

## 2. Materials and Methods

**Figure 1:**
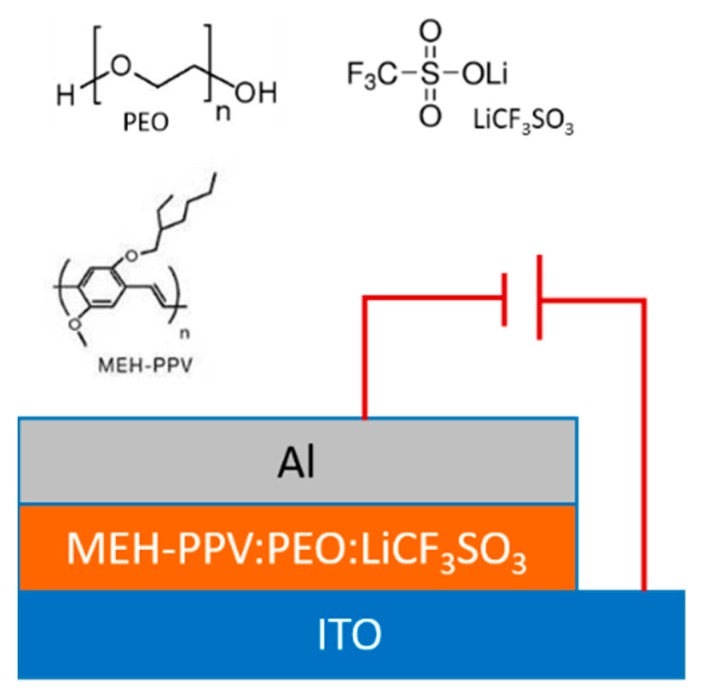
The chemical structures of these materials and the schematic view of the light-emitting electrochemical cells (LEC) device.

The LEC devices were prepared on cleaned ITO pattern glass substrate inside the glove box. The active polymer poly[5-(2′-ethylhexyloxy)-2-methoxy-1,4-phenylene vinylene] (MEH-PPV), the ion-transport materials, PEO and salt lithium trifluoromethanesulfonate (LiCF_3_SO_3_) were purchased from Sigma-Aldrich Inc., St. Louis, MO, USA, and were used as received. The chemical structures of these materials are shown in Figure 1. Firstly, three solutions of 8 mg/mL concentration were prepared: MEH-PPV was dissolved in chloroform, and PEO and LiCF_3_SO_3_ were dissolved separately in cyclohexanone at 50 °C by stirring for 18 h. Secondly, a blended solution was prepared by mixing the three solutions in a mass ratio of MEH-PPV/PEO/LiCF_3_SO_3_ = 8:8:1, and was then stirred on a magnetic hot plate at 50 °C for 2 h [19]. Thirdly, the clear solution mixture was spin-coated immediately onto an ITO substrate for 40 s at 1400 rpm. Then, the samples were transferred into a vacuum chamber for 10 min and heated at 88 °C on the hot plate to remove the residual solvent. Finally, an 80 nm thick aluminum top electrode was thermal-evaporated using mask at room temperature at about 1 × 10^−4^ Pa, with the Al evaporation rate of about 1–2 Å/s. Both the evaporation rate and the film thickness were determined by a quartz crystal thickness monitor (Sigma SQM-160, Inficon Inc., East Syracuse, NY, USA). The active area was about 3 mm × 3 mm. Before deposition, the substrates were successively cleaned using detergent, deionized water, isopropanol and acetone, then an UV-ozone treatment was performed. The above mass ratio and experimental procedure was similar to those in the reported references for LEC preparation [10,19]. A salt concentration that is too high resulted in the phase separation and foggy solution morphology, however too low salt content led to an insufficient ion concentration and a longer response time [20,21].

The device measurements were performed in a close-cycle cryostat with variable temperatures that were placed in a magnetic field. The magnetic field was provided by an electromagnet, and was applied parallel to the plane of the LEC sample. The luminance-current-voltage measurement was carried out using a Keithley 238 SourceMeter (Keithley Instruments Inc., Cleveland, OH, USA) and a silicon photodetector (Photoelectric Instrument Factory of Beijing Normal Unviersity, Beijing, China) connected to a Keithley 2000 Multimeter (Keithley Instruments Inc., Cleveland, OH, USA). The devices were driven at a constant voltage using a Keithley 238 SourceMeter, the electroluminescence (EL) were measured while sweeping B. The MEL were defined as ∆EL/EL = (EL(B) − EL(B = 0))/EL(B = 0).

## 3. Results and Discussion

**Figure 2:**
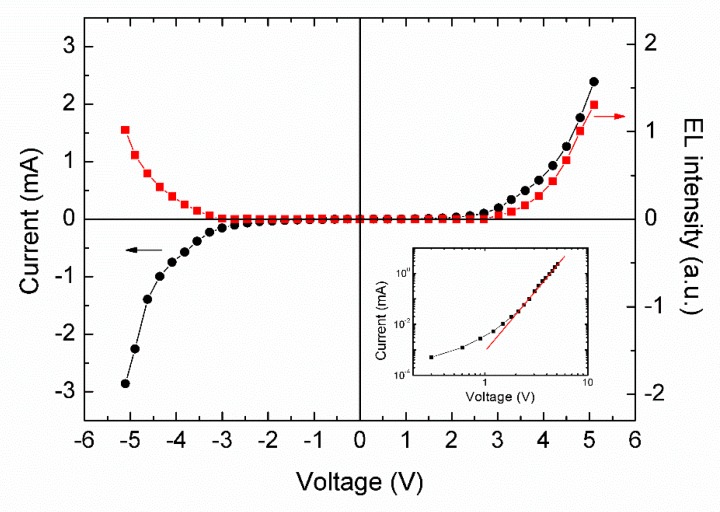
Luminance–current–voltage curves of LEC devices at 250 K, and the fitting results according to the power law are shown in the lower right inset.

Figure 2 presents the luminance–current–voltage curves of the LEC devices under both forward and reverse bias conditions at 250 K. It shows the typical bi-polar characteristics of LECs, with a roughly bilateral symmetric luminance–voltage curve and central symmetric current–voltage curve, which is obviously different from the diodelike behavior of conventional OLEDs. The threshold voltages for both forward-scan (sweeping from 0 to 5 V) and reverse-scan (sweeping from 0 to −5 V) were about 2.5 V. The threshold voltage is close to the band gap of MEH-PPV (2.1 eV). As is shown in the inset of Figure 2, the I-V characteristics of the device can be described by the power law of I∝V^m+1^ (m = 3.7) in the high-voltage region (above 2 V), which is the characteristic for the trapped-charge-limited current mode, indicating the presence of traps in the admixed organic layer [22].

According to the electrochemical doping model, upon the applied voltage, the oxidation and reduction of polymer MEH-PPV occurred from the interfaces near the electrodes with the moving of the ions, until the device reaches electrochemical equilibrium. After forming a stable p–i–n junction, the ionic current dropped to near zero, the actual current mainly originates from the transportation of electrons and holes. Under the applied field, the holes moved from the anode toward the cathode, and the electrons moved from the cathode toward the anode. These holes and electrons met in the active layer, forming the electron–hole pairs. Finally, the electron at LUMO and holes at HOMO recombined and radiatively decayed to the ground state [13]. The electrochemically induced p–i–n junction is dynamic and reversible, by changing the direction of the applied electric field, the direction of the p–n junction can be reversed, leading to the bi-polar characteristics of LECs.

**Figure 3:**
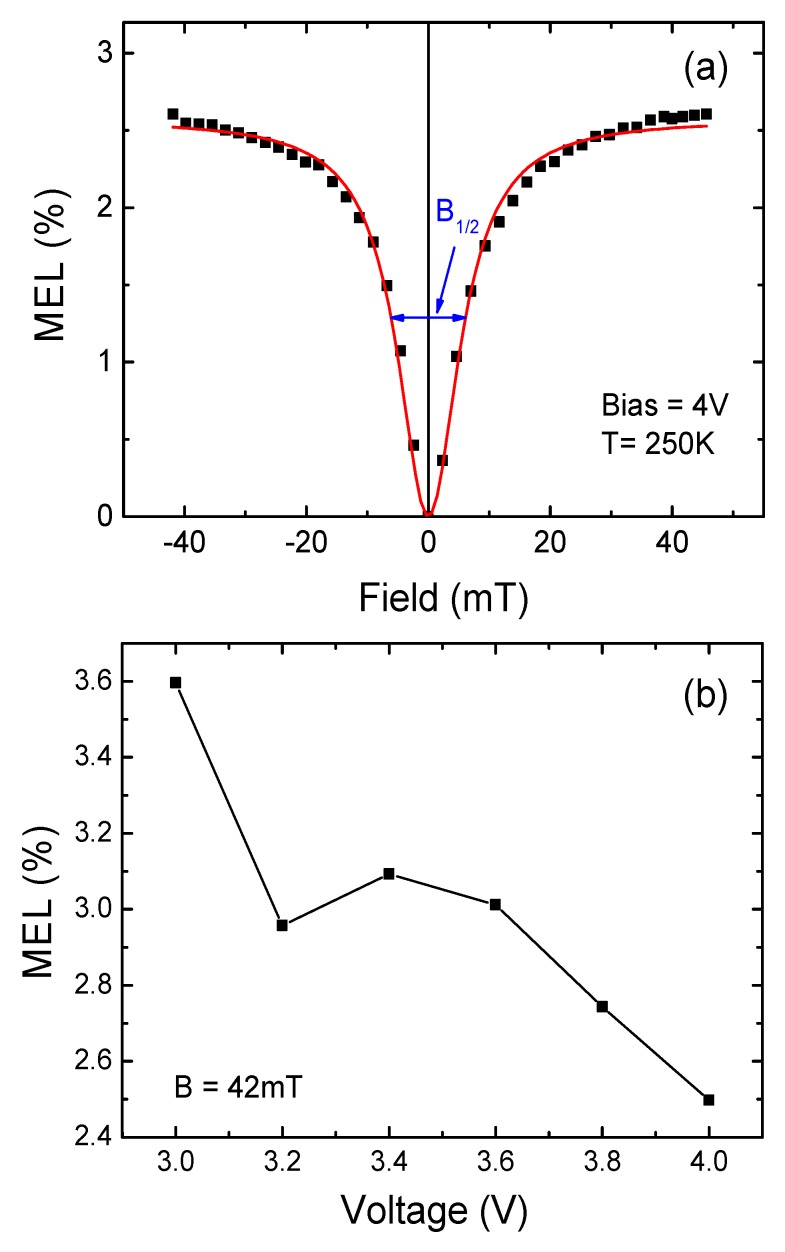
(**a**) The magnetic electroluminescence (MEL) response of the device under the bias voltage of 4 V at 250 K, the red solid lines show the Lorentzian line shape. (**b**) The MEL values as a function of bias voltage in the device under the applied field of 42 mT at 250 K.

Figure 3a shows the relationship between the MEL and the applied magnetic field of the LEC device at 250 K, the applied bias voltage is 4 V. The device shows positive MEL, about 2.5% (B = 42 mT), which is close to the MEL value of OLED devices [1,2,3,4]. The solid curve through the experimental data is a fit using an empirical law of “Lorentzian formula”: $\mathrm{MEL}\propto B^{2}/(B^{2}+B_{0}^{2})$, where B is the applied magnetic field and B_0_ is related to the hyperfine coupling strength with the value of 6.3 mT [23,24]. The half-field at half-maximum (HFHM) B_1/2_ that can be obtained from the curve, is 6.0 mT. A similar relationship between MEL and magnetic field has also been observed in OLEDs [23,24,25]. Recently, Hu et al. observed giant magnetic field effects in conventional electrochemical cells, where the field effect curve shapes are obviously different from our above results. They ascribe the phenomena to ion related process, such as the Lorentz force effects of mobile ions and the electrochemical reaction [26,27]. However, after forming stable p–i–n junction, the electrochemical redox and the redistribution of ions settled down and no longer engaged in the EL process of LECs. We thus conclude that the magnetic field effects in above LEC devices have similar origins to those in OLEDs, and show similar MEL characteristic of OLEDs. 

The empirical fitting relations of MEL was suggested that they can be related to processes involving spin flips due to the hyperfine interaction [24]. In the organic devices, hyperfine interaction plays a very important role, which could interconvert triplets into singlets and vice versa. In the presence of a magnetic field greater than the hyperfine coupling strength, it will reduce spin mixing of nearby carriers which results from the randomly oriented hyperfine fields, leading to a reduction in the intersystem conversion rate and an increase in the singlet/triplet ratio, finally leading to the enhancement of the electroluminescence. 

As shown in Figure 3b, the MEL responses decreased roughly with the increase of driving voltage, from 3.6% at 3 V to 2.5% at 4 V. The line shape of the MEL curves (not shown) with different bias voltages are similar, and can also be fitted with the above “Lorentzian formula”. In the LEC device, after the initial electrochemical doping, and with the increase of applied voltage, more electrons and holes inject from the electrodes and form excitons in the polymer active layer, which in turn causes a decrease of the e–h pair lifetime. Thus, only a part of excitons can contribute to the EL due to the shorter e–h pair lifetime, it is expected that fewer e–h pairs can be affected by the magnetic field and that the obtained values for MEL decrease with increasing voltage [28]. 

**Figure 4:**
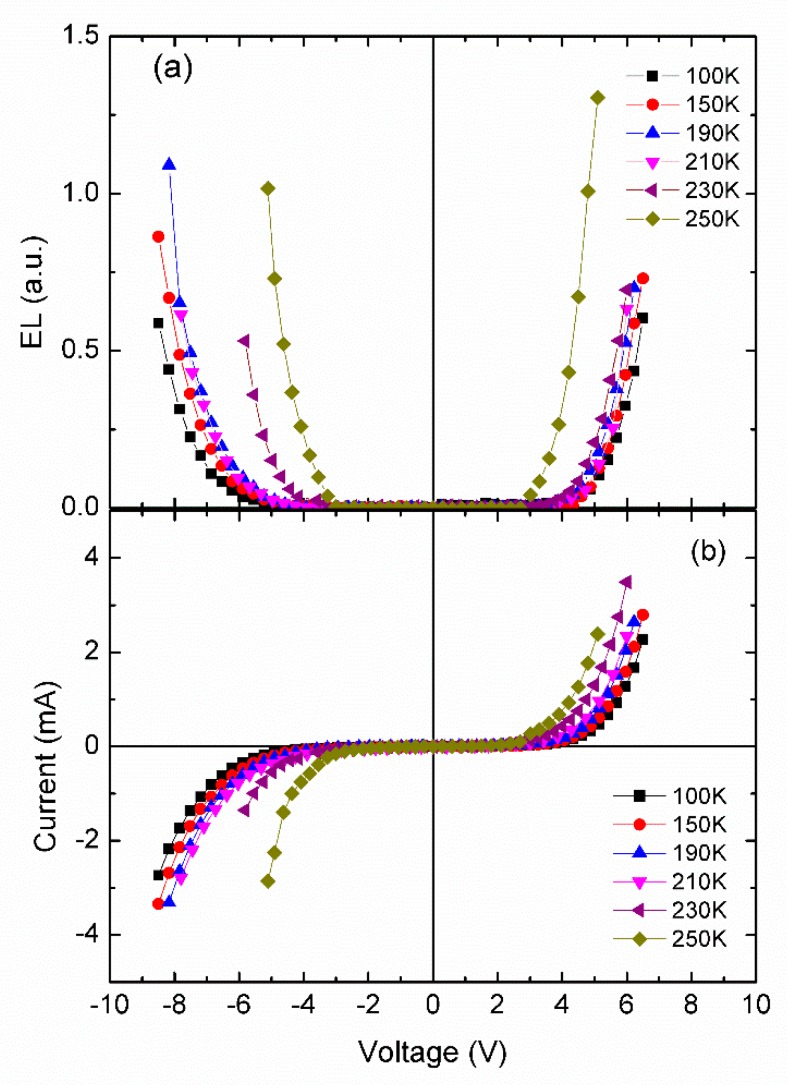
(**a**) The EL-V curves of LEC sample at different temperatures (from 250 K to 100 K); (**b**) The I-V curves of LEC sample at different temperatures (from 250 K to 100 K).

The influence in temperature on the luminance-current-voltage curves of the LEC device was investigated, with the decreasing temperature from 250 K to 100 K. As shown in Figure 4a,b, the luminance–current–voltage curves show the roughly bi-polar characteristics, however, the threshold voltages of reverse-scan are much higher than those of forward-scan at low temperature. As the temperature decreased, the positive and negative threshold voltage also increased. According to the free-volume theory of ionic transport in polymers, the ion mobility is proportional to $\exp\left[-B/\left(T-T_{c}\right)\right]$ [18]. With a decrease in temperature, the mobility of ions dramatically drops, both charge carrier injection and charge carrier transport become less efficient, resulting in the longer response time and higher threshold voltages at lower temperature. 

As we know, due to the low mobility of ions at low temperature, the charging history will influence the distribution of ions and EL properties for LEC devices. For comparison, we cooled a pristine LEC sample down directly from room temperature to 100 K without bias voltage, which is different from the above measurement sequence. At 100 K, it takes a very long charging period (about 3 hours) to lighten the LEC device under a higher bias voltage of 10.2 V with the poor EL performance and operating lifetime. 

**Figure 5:**
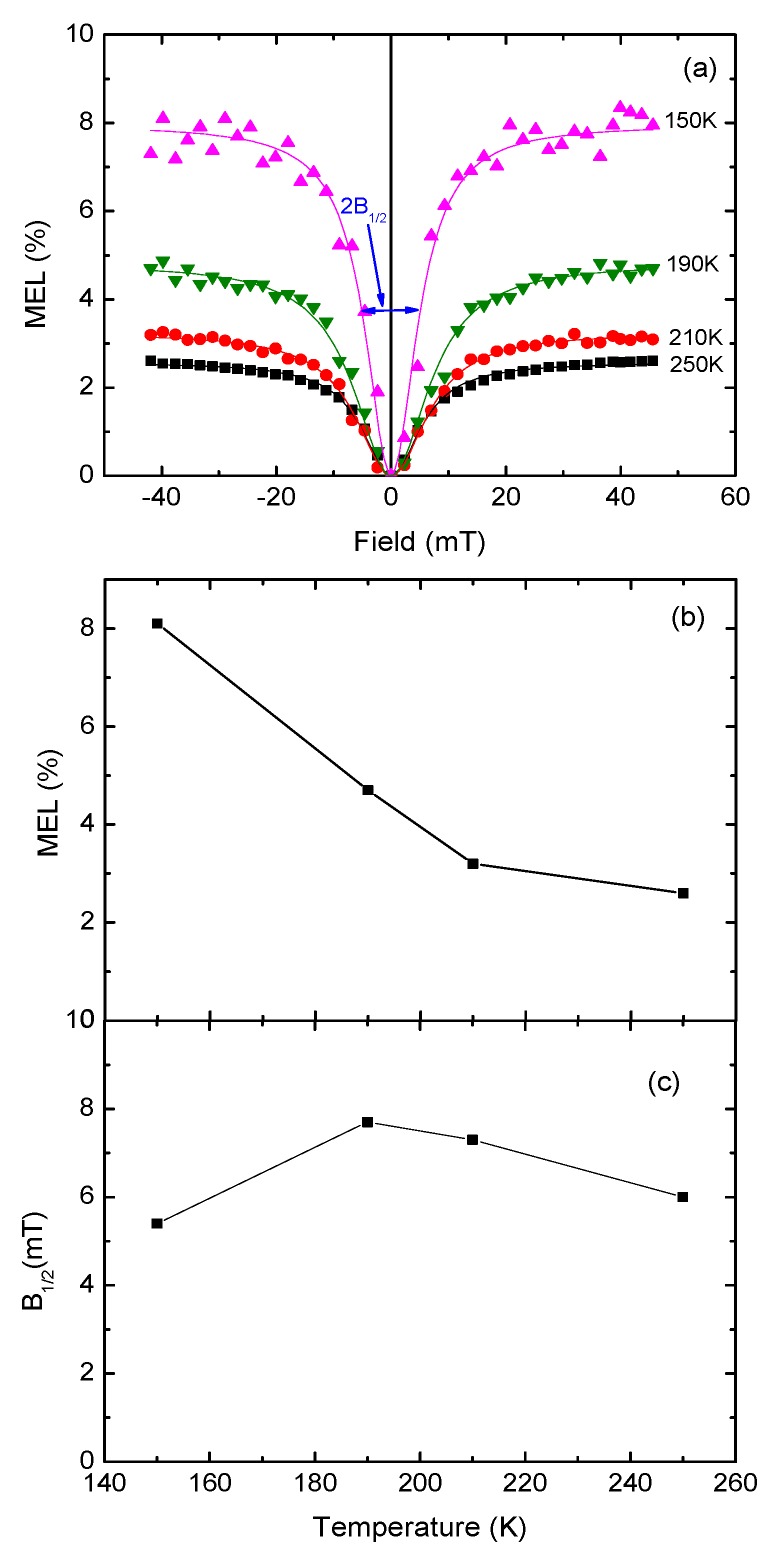
(**a**) The MEL curves of LEC sample at different temperatures (from 250 K to 150 K); (**b**) the MEL as a function of temperature in the LEC sample; (**c**) the temperature dependencies of the parameters B_1/2_ for the MEL response, extracted from (**a**).

Figure 5 shows MEL curves with the bias voltage of 4 V recorded at different temperatures from 250 K to 150 K. It was noted that, since the threshold voltage was higher than 4 V at 100 K, the MEL curve of 100 K hadn’t been obtained. As is seen in Figure 5a, the EL intensity of the LEC sample was enhanced with the increase of the magnetic field at different temperatures, showing the similar shape of MEL curves. As shown in Figure 5b, starting at 250 K, the MEL value rose accordingly with the drop in temperature, then showed a maximum of about 8% at 150 K. The temperature dependence of MEL in the LEC was similar to that in the MEH-PPV based OLEDs, but was more remarkable than the latter [7]. In Figure 5c, B_1/2_ increased gradually with the temperature decreasing from 250 K to 190 K, then dropped to 150 K, which is partly inconsistent with the temperature dependence of MEL. It is suggested that the magnetic field effects (MFE) mechanism of LEC is more complicated and needs further study.

As we know, with decreasing temperature the charge carrier mobility is reduced and the lifetime of the e–h pairs increases. Consequently, the number of e–h pairs that are affected by the magnetic field is assumed to increase, which presumably causes the increasing change of MEL [28]. Additionally, the interaction of charge carriers with triplet states possibly leads to the quench of triplet excitons. With a decrease in temperature, the quench process of triplet excitons will weaken accordingly due to the decreasing injected carriers, thus increasing the number of the singlet excitons through the magnetic field dependent intersystem crossing from triplet to singlet state, finally resulting in the enhancement of MEL [29]. 

## 4. Conclusions

In summary, we fabricated a LEC device with the structure of ITO/MEH-PPV:PEO:LiCF_3_SO_3_/Al and observed positive magnetic field effects on electroluminescence in the sample at various temperatures with the MEL value of about 2.5% (B = 42 mT, 250 K), showing a Lorentzian line shape. The voltage and temperature dependence of MEL were also investigated, with the increase of voltage and temperature, the MEL increased accordingly. The possible mechanism was discussed.
